# Unifying scrambling, thermalization and entanglement through measurement of fidelity out-of-time-order correlators in the Dicke model

**DOI:** 10.1038/s41467-019-09436-y

**Published:** 2019-04-05

**Authors:** R. J. Lewis-Swan, A. Safavi-Naini, J. J. Bollinger, A. M. Rey

**Affiliations:** 1 JILA, NIST and Department of Physics, University of Colorado, Boulder, CO 80309 USA; 2 Center for Theory of Quantum Matter, University of Colorado, Boulder, CO 80309 USA; 3 NIST, Boulder, CO 80305 USA

**Keywords:** Quantum information, Quantum simulation, Statistical physics, thermodynamics and nonlinear dynamics

## Abstract

Scrambling is the process by which information stored in local degrees of freedom spreads over the many-body degrees of freedom of a quantum system, becoming inaccessible to local probes and apparently lost. Scrambling and entanglement can reconcile seemingly unrelated behaviors including thermalization of isolated quantum systems and information loss in black holes. Here, we demonstrate that fidelity out-of-time-order correlators (FOTOCs) can elucidate connections between scrambling, entanglement, ergodicity and quantum chaos (butterfly effect). We compute FOTOCs for the paradigmatic Dicke model, and show they can measure subsystem Rényi entropies and inform about quantum thermalization. Moreover, we illustrate why FOTOCs give access to a simple relation between quantum and classical Lyapunov exponents in a chaotic system without finite-size effects. Our results open a path to experimental use FOTOCs to explore scrambling, bounds on quantum information processing and investigation of black hole analogs in controllable quantum systems.

## Abstract (web summary)

The scrambling of quantum information in a many-body system leads to the emergence of statistical mechanics and chaotic behaviour. Here the authors establish quantitative relationships between experimentally-measureable correlators, the Rényi entropy and Lyapunov exponents in the Dicke model.

## Introduction

Recent studies have shown that isolated many-body quantum systems, under unitary time evolution, can become highly entangled and thus thermalize. This understanding has led to insights as to how statistical mechanics emerges in closed quantum systems^1–3^. Moreover, the relevance of entanglement as a resource for quantum information processing, quantum communication and metrology has stimulated cross-disciplinary efforts to quantify and characterize entanglement. Experimental progress in controlling clean, highly isolated, and fully tunable quantum systems, where entanglement can be measured, have resulted in radical advances in this direction. However, such measurements have been restricted to few body systems, including arrays of 6 × 2 bosonic atoms^4^, three superconducting qubits^5^, and systems of $\lesssim 20$ trapped ions^6,7^. The model we study here and the measurements we propose can be implemented in trapped ions with more than 100 spins.

**Fig. 1:**
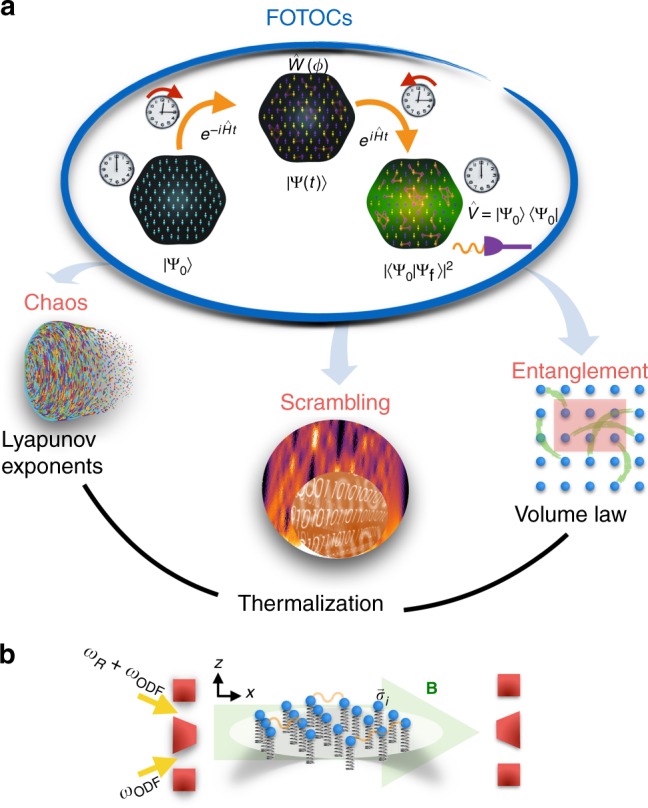
Unifying chaos, scrambling, entanglement and thermalization through the measurement of fidelity out-of-time-order correlators (FOTOCs). **a** Scheme: an initial state, |ψ_0_〉 is evolved under an interacting Hamiltonian $\hat {\mathrm{H}}$ for a time *t*. Inverting the sign of $\hat {\mathrm{H}}$ and evolving again for time *t* to the final state |ψ_f_〉, implements the many-body time-reversal, which ideally takes the system back to the initial state |ψ_0_〉. If a perturbation $\hat W(\phi )$ is inserted between the two halves of the time evolution and the many-body overlap with the initial state is measured at the end of the protocol, ${\hat{V}} = \left| {{\Psi} _0} \right\rangle \left\langle {{\Psi} _0} \right|$, then a special type of fidelity OTOC (FOTOC) is implemented. **b** The Dicke model is engineered in a Penning trap ion crystal by applying a pair of lasers, resonant only with the center-of-mass mode, to generate the spin−phonon interaction and resonant microwaves to generate the transverse field

Concurrently, out-of-time-order correlations (OTOCs)^8–13^

1 $$F(t) = \langle \hat W^\dagger (t)\hat V^\dagger \hat W(t)\hat V\rangle ,$$

have been identified as measures of the dynamics of quantum information scrambling. Here, $\hat W(t) = e^{i\hat {\mathrm{H}}t}\hat We^{ - i\hat {\mathrm{H}}t}$, with $\hat {\mathrm{H}}$ a quantum many-body Hamiltonian, and $\hat W$ and $\hat V$ two initially commuting and unitary operators. While OTOCs can be computed with respect to any (possibly mixed) state, here we focus on the case where the initial state of the system is pure. The quantity ${\mathrm{Re}}[F(t)] = 1 - \langle [\hat V^\dagger ,\hat W^\dagger (t)][\hat W(t),\hat V]\rangle /2$ encapsulates the degree that $\hat W(t)$ and $\hat V$ fail to commute at later times due to the time evolution of $\hat W$ under $\hat {\mathrm{H}}$. (If $\hat V$ is not unitary but a projector, e.g. $\hat V\hat V^\dagger = \hat V$ and $\hat V$ commutes with the density matrix of the initial state, then ${\mathrm{Re}}[F(t)] = 1 - \langle [\hat V^\dagger ,\hat W^\dagger (t)][\hat W(t),\hat V]\rangle$.) The fastest scramblers^8–10,14^, such as black holes, feature an exponential growth of scrambling which manifests as $1 - {\mathrm{Re}}[F(t)]\sim e^{\lambda _{\mathrm{Q}}t}$. Here, *λ*_Q_ is the quantum Lyapunov exponent that serves as a proxy for quantum chaos. Regardless of the OTOCs’ apparent complexity^13,15–17^, the capability to perform many-body echoes (see Fig. 1) in current experiments^18–21^ has opened a path for the experimental investigation of quantum scrambling; however, so far those have not probed quantum chaos or fast scrambling.

Here we show that fidelity out-of-time-order correlators (FOTOCs), a specific family of fidelity out-of-time-order correlators, which set $\hat V$ to be a projector on the initial state, can provide profound insight on scrambling behavior. We explicitly compute FOTOCs in the in the Dicke model^22^, an iconic model in quantum optics, and illustrate how FOTOCs elucidate theoretical connections between scrambling, volume-law Rényi entropy (RE) and thermalization, while linking quantum and classical chaos (Fig. 1a). Additionally, we discuss how one can probe these connections readily in experiments.

## Results

### Model

The Dicke model (DM)^22^ describes the coupling of a single large spin and a harmonic oscillator and has been recently implemented in atomic^23–26^ and trapped ion setups^27^. The Hamiltonian of the DM is given by

2 $$\hat {\mathrm{H}}_{\mathrm{D}} = \frac{{2g}}{{\sqrt N }}\left( {\hat a + \hat a^\dagger } \right)\hat S_z + \delta \hat a^\dagger \hat a + B\hat S_x,$$

where *B* characterizes the strength of the transverse field, *δ* the detuning of the bosonic mode from the driving field with strength *g* that generates the spin−boson coupling. Here, *g*, *δ*, *B* ≥ 0. The operator $\hat a$ ($\hat a^\dagger$) is the bosonic annihilation (creation) operator of the mode, and $\hat S_\alpha = \mathop {\sum}\nolimits_{j = 1}^N {\hat \sigma _j^\alpha } 2$ are collective spin operators with $\hat \sigma _j^\alpha$ (*α* = *x*, *y*, *z*) the Pauli matrices for the *j*th spin-1/2.

### Connections between scrambling dynamics and chaos

**Fig. 2:**
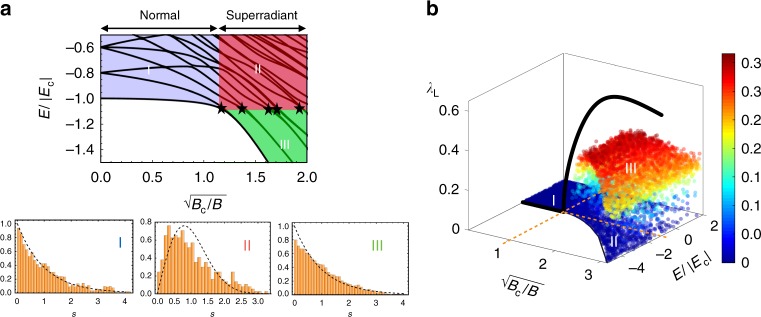
Characterization of classical and quantum chaos in the Dicke model. **a** Phase diagram of the Dicke model. At zero temperature it exhibits a quantum phase transition between a normal to a superradiant phase, at *B* = *B*_c_. A line of excited energy quantum phase transitions (ESQPTs) occurs at the critical energy *E*_c_ = −*BN*/2, signaled by singularities in the energy level structure (indicated by stars). Note that for figure clarity we have used a small system *N* = 20 resulting in the small deviation of the ESQPTs from *E*_c_ = −*BN*/2. The ESQPTs are accompanied by a change in the level statistics which we denote by (I)−(III) (note that no eigenstates exist in the unlabeled white region). For (II) and (III) the spectrum is divided into low and high energy parts, separated by the ESQPT at *E* = *E*_c_, from which the statistics *P*(*s*), where *s* is the level spacing, are computed separately. (I) exhibits Poissonian statistics (regular regime), while (II) displays statistics similar to a Wigner−Dyson distribution indicative of level repulsion and quantum chaos, and (III) exhibits a mixture of both. The numerical parameters are *g*/(2*π*) = 0.66 kHz and *δ*/(2*π*) = 0.5 kHz. **b** Lyapunov exponents for the mean-field dynamics of an ensemble of random states sorted by normalized mean-field energy *E*/|*E*_c_| with *E*_c_ = −*BN*/2, as a function of the field $\sqrt {B_{\mathrm{c}}/B}$ relative to critical field *B*_c_ = 4*g*^2^/*δ*. A crossover between regular (*B* > *B*_c_) and chaotic dynamics (*B* < *B*_c_) characterized by $\lambda \simeq 0$ and *λ*_L_ > 0 respectively, occurs at *B* = *B*_c_. For *B* > *B*_c_ and energies $E \ \lesssim \ E_{\mathrm{c}}$ the dynamics becomes increasingly regular. Source data are provided as a Source Data file

Even when restricted to the Dicke manifold, i.e. states with *S* = *N*/2, with *S*(*S* + 1) the eigenvalue of the total spin operator $\hat S^2 = \hat S_x^2 + \hat S_y^2 + \hat S_z^2$, this model exhibits rich physics (see Fig. 2a). At zero temperature, *T* = 0, the DM features a quantum phase transition (QPT) as the system crosses a critical field *B*_c_ = 4*g*^2^/*δ*. For *B* > *B*_c_ (normal phase), the ground-state is described by spins aligned along the transverse field and a bosonic vacuum. For *B* < *B*_c_ (superradiant phase), the ground-state is ferromagnetic, $\langle |\hat S_Z|\rangle \sim N/2$, and characterized by macroscopic occupation of the bosonic mode (Fig. 2a). Furthermore, in the superradiant phase (*B* < *B*_c_), the DM features a family of excited-state quantum phase transitions (ESQPTs). The ESQPTs are signaled by singularities in the energy-level structure and a change in the spectral statistics^28–31^ at a critical energy *E*_c_ = −*BN*/2 that coincides with the ground-state energy of the normal phase. Figure 2a shows how the nearest-neighbor spacing distribution *P*(*s*), where *s* is a normalized distance between two neighboring energy levels, features a different character on either side of *E*_c_. For *E* > *E*_c_ the spectral statistics are similar to the Wigner−Dyson distribution $P_{\mathrm{W}}(s) = \pi s/2{\mathrm{exp}}( - \pi s^2/4)$, which in random-matrix theory describes a chaotic system. For *E* < *E*_c_ the shape of the histograms is neither Wigner−Dyson nor Poissonian *P*_P_(*s*) = exp(−*s*). The latter characterizes level statistics of non-ergodic systems, and is observed in the normal phase. While the deviations from clear Wigner−Dyson or Poissonian statistics in regimes II and III are attributable to finite-size effects^28^, we emphasize that even for this small system they clearly show a stark contrast in the degree of level repulsion, which is a qualitative signature of quantum chaos.

Similar features appear in the classical dynamics of the DM^30,32–35^, manifested in the different behavior of trajectories in phase-space computed from the mean-field equations of motion for: ${\tilde{\mathbf{x}}} = (\langle \hat S_x\rangle ,\langle \hat S_y\rangle ,\langle \hat S_z\rangle ,\alpha _{\mathrm{R}},\alpha _{\mathrm{I}})$, where $\langle \ldots \rangle$ denotes the expectation values, and *α*_R(I)_ is the real (imaginary) part of $\langle \hat a\rangle$. In the superradiant phase and for mean-field energies $E \ > \ E_{\mathrm{c}}$, two trajectories initially separated by $\Delta {\tilde{\mathbf{x}}}(0)$ in phase-space diverge as $|\Delta {\tilde{\mathbf{x}}}(t)|\sim |\Delta {\tilde{\mathbf{x}}}(0)|e^{\lambda _{\mathrm{L}}t}$ at sufficiently long times^36^. The exponential growth, associated with a positive Lyapunov exponent *λ*_L_ > 0, diagnoses chaos in a classical system. In Fig. 2b we show the maximal Lyapunov exponent for an ensemble of random initial product states as a function of the transverse field and the normalized mean-field energy *E*/*E*_c_ (see Methods). For *E* < *E*_c_ in the superradiant phase (*B* < *B*_c_) and all energies in the normal phase (*B* > *B*_c_), the Lyapunov exponent is small or zero, consistent with the Poissonian character of the quantum-level statistics in this parameter regime^34,35^. For *E* > *E*_c_ and *B* < *B*_c_ a positive exponent is found signaling chaos. Note that the state $|\Psi _0^{\mathrm{c}}\rangle = |( - N/2)_x\rangle \otimes |0\rangle$, where $\hat S_x|( - N/2)_x\rangle = \left( { - N/2} \right)|( - N/2)_x\rangle$, lies exactly at the ESQPT critical energy, $\langle \Psi _0^{\mathrm{c}}|\hat H_{\mathrm{D}}|\Psi _0^{\mathrm{c}}\rangle = E_{\mathrm{c}}$, and possesses the largest classical *λ*_L_ (see Fig. 2).

In quantum systems OTOCs may serve as a diagnostic for quantum chaos. However, such diagnosis has proved difficult, since any exact numerical treatment is only possible in small systems, where many-body observables saturate quickly at the Ehrenfest time given by $\lambda _{\mathrm{Q}}t^ \ast \sim {\mathrm{log}}N$, at which the quantum information is thoroughly lost to a “local” observer. Here we demonstrate that we can overcome this limitation and compute OTOCs for macroscopic systems if, for a Hermitian operator $\hat G$, one restricts $\hat W_G = e^{i\delta \phi \hat G}$ to be a sufficiently small perturbation $\left( {\delta \phi \ll 1} \right)$ and sets $\hat V$ to be a projection operator onto a simple initial state |ψ_0_〉, i.e. $\hat V = \hat \rho (0) = |\Psi _0\rangle \langle \Psi _0|$. This is because in the perturbative limit $\delta \phi \ll 1$, this particular type of fidelity OTOC (FOTOC)^18,19^, ${\cal{F}}_G(t,\delta \phi ) \equiv \langle \hat W_G^\dagger (t)\hat \rho (0)\hat W_G(t)\hat \rho (0)\rangle$ (such that for a pure state ${\cal{F}}_G(t) \equiv |\langle \psi _0|e^{i\hat {\mathrm{H}}t}e^{i\delta \phi \hat G}e^{ - i\hat {\mathrm{H}}t}|\psi _0\rangle |^2$) reduces to^37^

3 $$1 - {\cal{F}}_G(t,\delta \phi ) \approx \delta \phi ^2(\langle \hat G^2(t)\rangle - \langle \hat G(t)\rangle ^2) \equiv \delta \phi ^2{\mathrm{var}}[\hat G(t)],$$

where ${\mathrm{var}}[\hat G(t)]$ is the variance of $\hat G$. This relation establishes a connection between the exponential growth of quantum variances and quantum chaos, enables us to visualize the scrambling dynamics of a quantum system using a semi-classical picture^38^ and to map the FOTOC to a two-point correlator which can be computed using well-known phase-space methods, such as the truncated Wigner approximation (see Methods)^39,40^. We observe perfect agreement between the exact dynamics of the FOTOC with the associated variance, ${\mathrm{var}}(\hat G)$ for sufficient small *δϕ*, enabling us to use phase-space methods to compute the FOTOCs in a parameter regime inaccessible to exact numerical diagonalization where exponential scrambling can be clearly identified.

Moreover, it provides a link between the FOTOCs and the quantum Fisher information (QFI)^19,41–43^, as the variance of $\hat G$ is proportional to the QFI of a pure state, whilst for a mixed state the variance gives a lower bound on the QFI. Note that in the latter case FOTOCs are defined by replacing $\hat V$ by the initial density matrix $|\Psi _0\rangle \langle \Psi _0| \to \hat \rho _0$, and expectation values are computed by appropriate traces. The QFI quantifies the maximal precision with which a parameter *δϕ* in the unitary of $\hat W$ can be estimated using an interferometric protocol with an input quantum state |ψ(*t*)〉, while simultaneously serving as a witness to multipartite entanglement^19,44–46^.

**Fig. 3:**
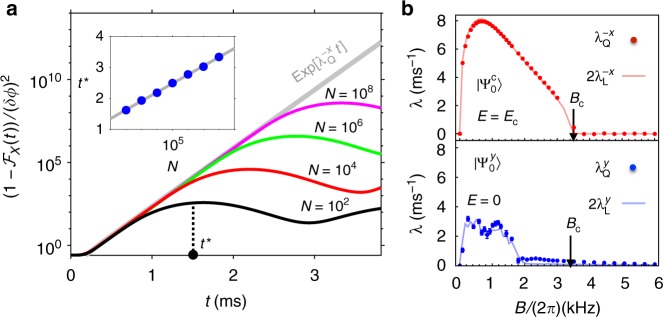
Signatures of classical chaos in quantum FOTOCs. **a** Initial exponential growth of the FOTOC, $[1 - {\cal{F}}_X(t)]/(\delta \phi )^2$ and the initial state $|\Psi _0^{\mathrm{c}}\rangle = |( - N/2)_x\rangle \otimes |0\rangle$ (see Supplementary Note 1 for examples of exponential growth in other states). We assume $\delta \phi \ll 1/N$ such that we may equivalently use ${\mathrm{var}}(\hat X) \simeq [1 - {\cal{F}}_X(t)]/(\delta \phi )^2$ for the plotted data. The scrambling time t^*^ is defined by the saturation of the FOTOC, which we extract from the first maximum and plot in the inset (blue data). We find $t^ \ast \sim a_0 + {\mathrm{log}}(N)/\lambda _{\mathrm{Q}}$ with *a*_0_ a fit parameter (gray line). **b** Lyapunov exponent, *λ*, as a function of transverse field: Quantum *λ*_Q_ (red markers) and classical 2*λ*_L_ (solid lines). Superscript notation of the exponents denotes the initial polarization of the chosen coherent spin state. Top panel for $|\Psi _0^{\mathrm{c}}\rangle$, the same state as (**a**), and bottom for $|\Psi _0^y\rangle \equiv |( - N/2)_y\rangle \otimes |0\rangle$, here *N* = 10^4^ particles. In both plots we observe $\lambda _{\mathrm{Q}} \simeq 2\lambda _{\mathrm{L}}$. Error bars for *λ*_Q_ are a 95% confidence interval from an exponential fitted to the numerical data. Coupling *g* and detuning *δ* are same as Fig. 2. In (**a**) *B*/(2*π*) = 0.7 kHz (*B*/*B*_c_ = 0.2). Source data are provided as a Source Data file

In Fig. 3a we plot the FOTOCs of a small perturbation using $\hat G = \hat X = \frac{1}{2}(\hat a + \hat a^\dagger )$ starting with $|\Psi _0\rangle = |\Psi _0^{\mathrm{c}}\rangle$. In the superradiant phase we observe that after a short time of slow dynamics, $t_\lambda \sim \lambda _{\mathrm{Q}}^{ - 1}$, the FOTOCs feature an exponential growth $\sim e^{\lambda _{\mathrm{Q}}t}$, before saturating at $t^ \ast \sim {\mathrm{log}}\ N$ (see inset). The quantum exponent is found to be independent of system size *N*. For this initial state, and all the product states we have investigated numerically (Supplementary Note 1), we have observed that $\lambda _{\mathrm{Q}} \simeq 2\lambda _{\mathrm{L}}$, as shown in Fig. 3b. Indeed, for any $\hat G$ that corresponds to a linear function of the classical phase-space variables (see Methods and Supplementary Note 1), the quantum exponent should be related to the classical Lyapunov exponent by this relation. A similar factor of two relating the classical and quantum exponents has previously been observed in refs. ^37,47–49^. This correspondence can be explained by semi-classical arguments (see Methods), and the numeric prefactor is attributable to the definition of the classical Lyapunov exponent in terms of a distance in phase-space, while the FOTOC reduces to the quantum variance.

### FOTOCs as a probe of entanglement and quantum thermalization

We now move beyond the semi-classical arena and explore connections between FOTOCs and entanglement entropy. In a closed system *S* the second-order Rényi entropy, RE, $S_2(\hat \rho _A) = - {\mathrm{logTr}}(\hat \rho _A^2)$ measures the entanglement between a subsystem *A* and its complement *A*_c_ = *S* − *A*, with $\hat \rho _A$ the reduced density matrix of *A* after tracing over *A*_c_. Although scrambling and entanglement buildup are closely connected, they are not the same. Nevertheless, a formal relationship between the OTOCs and $S_2(\hat \rho _A)$ exists^11^, which requires averaging OTOCs over a complete basis of operators of the system subsystem *A*. Based on this relation, measuring RE via OTOCs appears as challenging as directly measuring $S_2(\hat \rho _A)$. However, this is not always the case. We will show that for collective Hamiltonians, such as the DM, there is a simple correspondence between the Fourier spectrum of FOTOCs and the RE, which facilitates experimental access to $S_2(\hat \rho _A)$ via global measurements and collective rotations.

To illustrate the connection we first write the density matrix of the full system in a basis spanned by the eigenstates of the spin operator $\hat S_{\mathbf{r}} \equiv (e_{\mathbf{r}} \cdot S),$ where e_**r**_ is a unit vector in the Bloch sphere, satisfying $\hat S_{\mathbf{r}}|m_{\mathbf{r}}\rangle = m_{\mathbf{r}}|m_{\mathbf{r}}\rangle$, and $\hat n|n\rangle = |n\rangle$ the mode number operator $\hat n = \hat a^\dagger \hat a$, i.e. $$\hat \rho = \mathop {\sum}\nolimits_{\scriptstyle n,n\prime \atop\\ \scriptstyle m_{\mathbf{r}},m_{\mathbf{r}}^\prime } {\rho _{m_{\mathbf{r}}^\prime ,m_{\mathbf{r}}}^{n\prime ,n}} |n\prime \rangle \langle n| \otimes |m_{\mathbf{r}}^\prime \rangle \langle m_{\mathbf{r}}|$$. We adopt a convention for the coefficients of the density matrix elements where superscripts are associated with the bosonic mode, and subscripts with the spin. In this basis the density matrix can be divided into blocks, $\hat \rho = \mathop {\sum}\nolimits_M {\hat \rho _M^{\hat S_{\mathrm{r}}}}$ with $$\hat \rho _M^{\hat S_{\mathbf{r}}} = \mathop {\sum}\nolimits_{\scriptstyle n,n\prime \atop\\ \scriptstyle m_{\mathbf{r}}} {\rho _{m_{\mathbf{r}} + M,m_{\mathbf{r}}}^{n\prime ,n}} |n\prime \rangle \langle n| \otimes |m_{\mathbf{r}} + M\rangle \langle m_{\mathbf{r}}|$$, in such a way that $\hat \rho _M^{\hat S_{\mathbf{r}}}$ contains all coherences between states with spin eigenvalues that differ by *M*. A similar decomposition can be performed in terms of the bosonic coherences as $\hat \rho = \mathop {\sum}\nolimits_M {\hat \rho _M^{\hat n}}$ with $$\hat \rho _M^{\hat n} = \mathop {\sum}\nolimits_{\scriptstyle n \\ \scriptstyle m_{\mathbf{r}},m_{\mathbf{r}}^\prime } {\rho _{m_{\mathbf{r}}^\prime ,m_{\mathbf{r}}}^{n + M,n}} |n + M\rangle \langle n| \otimes |m_{\mathbf{r}}^\prime \rangle \langle m_{\mathbf{r}}|$$. Associated with this representation one can define the so-called multiple quantum intensities $I_M^{\hat G} = {\mathrm{Tr}}[\hat \rho _{ - M}^{\hat G}\hat \rho _M^{\hat G}]$. Of particular interest for us are the $I_0^{\hat G}$ components which are “incoherent” with respect to $\hat G$.

The intensities $I_M^{\hat G}(t)$ can be accessed experimentally from FOTOCs via the relation ${\cal{F}}_G(t,\phi ) = \mathop {\sum}\nolimits_M I_M^{\hat G}(t)e^{ - iM\phi }$^18,19,50–52^ by choosing $\hat W_G(\phi ) = e^{ - i\phi \hat G}$ and $\hat G = \hat S_{\mathbf{r}}$ or $\hat G = \hat n$, i.e. collective spin or boson rotations respectively. In terms of the $I_M^{\hat G}(t)$ the entanglement between the spins and the phonons characterized by the purity $${\mathrm{Tr}}\left[ {\hat \rho _{\mathrm{{ph}}}^2} \right] = \mathop {\sum}\nolimits_{\scriptstyle n,n\prime \atop\\ \scriptstyle m_{\mathbf{r}},m_{\mathbf{r}}^\prime } {\rho _{m_{\mathbf{r}},m_{\mathbf{r}}}^{n,n\prime }} \rho _{m_{\mathbf{r}}^\prime ,m_{\mathbf{r}}^\prime }^{n\prime ,n}$$ can be written as

4 $${\mathrm{Tr}}\left[ {\hat \rho _{{\mathrm{ph}}}^2(t)} \right] \equiv I_0^{\hat S_{\mathbf{r}}}(t) + I_0^{\hat n}(t) - D_{{\mathrm{diag}}}^{\hat S_{\mathbf{r}},\hat n}(t) + C_{{\mathrm{off}}}^{\hat S_{\mathbf{r}},\hat n}(t).$$

The terms $D_{{\mathrm{diag}}}^{\hat S_{\mathbf{r}},\hat n}(t)$ and $C_{{\mathrm{off}}}^{\hat S_{\mathbf{r}},\hat n}(t)$ are explicitly detailed in the Methods, but importantly $D_{{\mathrm{diag}}}^{\hat S_{\mathbf{r}},\hat n,}(t)$ is composed purely of the diagonal elements of $\hat \rho$ while $C_{{\mathrm{off}}}^{\hat S_{\mathbf{r}},\hat n}(t)$ contains information about coherences. During unitary evolution the characteristic dephasing time of the coherences is $t_{\mathrm{c}}\sim \lambda _{\mathrm{Q}}^{ - 1}$, which for scrambling systems is much faster than $t^ \ast \sim \lambda _{\mathrm{Q}}^{ - 1}{\mathrm{log}}\ N$. After *t*_c_ any remaining coherences are fully randomized and destructively interfere yielding $C_{{\mathrm{off}}}^{\hat S_{\mathbf{r}},\hat n} \to 0$. This feature, together with the fact that for those systems also the magnitude of $D_{{\mathrm{diag}}}^{\hat S_{\mathbf{r}},\hat n}$ becomes much smaller than $I_0^{\hat S_{\mathbf{r}}}$ and $I_0^{\hat n}$ as the density matrix spreads out over the systems degrees of freedom, allows us to approximate ${\mathrm{Tr}}\left[ {\hat \rho _{{\mathrm{ph}}}^2(t)} \right] \approx I_0^{\hat S_{\mathbf{r}}}(t) + I_0^{\hat n}(t)$. While at *t* < *t*_c_ these conditions are not necessarily satisfied, we still find that there can be a correspondence between the FOTOCs and RE by picking a state that is fully incoherent at time *t* = 0, $C_{{\mathrm{off}}}^{\hat S_{\mathbf{r}},\hat n}(0) = 0$. An example of such a state is $|\Psi _0^{\mathrm{c}}\rangle$ and $\hat G = \hat S_x$. This choice enforces the $C_{{\mathrm{off}}}^{\hat S_{\mathbf{r}},\hat n}$ term to remain small at short times. Moreover, for |Ψ_0_〉 we find it is also possible to access $S_2(\hat \rho _{{\mathrm{ph}}})$ via $I_0^{\hat S_{\mathbf{r}}}$ even in the regime *B* > *B*_c_, where no scrambling occurs. This is because the contributions from $I_0^{\hat n}$ and $D_{{\mathrm{diag}}}^{\hat S_{\mathbf{r}},\hat n}$ cancel and ${\mathrm{Tr}}\left[ {\hat \rho _{{\mathrm{ph}}}^2(t)} \right] \approx I_0^{\hat S_{\mathbf{r}}}$.

**Fig. 4:**
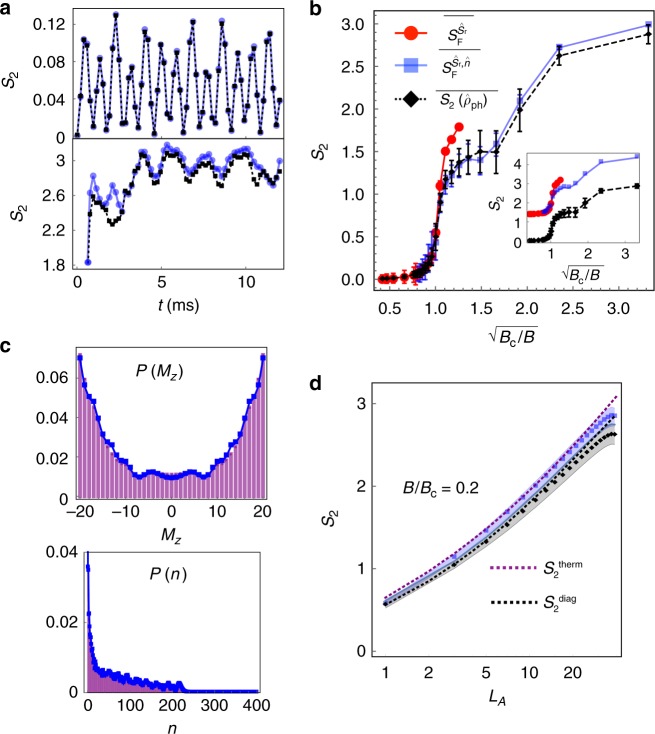
Using RE and FOTOCs to characterize chaos and thermalization in the Dicke model. **a** Time evolution of the spin−phonon RE $S_2(\hat \rho _{{\mathrm{ph}}})$ (black lines) for the initial state $|\Psi _0^{\mathrm{c}}\rangle = |( - N/2)_x\rangle \otimes |0\rangle$ with *B* > *B*_c_ (top) and *B* < *B*_c_ (bottom). The RE is tracked excellently by the FOTOC expressions (blue lines) $S_{\mathrm{F}}^{\hat S_{\mathbf{r}}} = - {\mathrm{log}}(I_0^{\hat S_{\mathbf{r}}})$ and $S_{\mathrm{F}}^{\hat S_{\mathbf{r}},\hat n} = - {\mathrm{log}}(I_0^{\hat S_{\mathbf{r}}} + I_0^{\hat n})$ respectively. Here, $\hat S_{\mathbf{r}}$ is chosen to minimize the coherence and diagonal terms in Eq. (4) (Supplementary Methods). **b** Long-time spin−phonon RE $S_2(\hat \rho _{{\mathrm{ph}}})$ as a function of transverse field. To remove finite-size effects and residual oscillations we plot a time-averaged value $\overline {S_2(\hat \rho _{{\mathrm{ph}}})}$ for 4 ms ≤  *t* ≤ 12 ms (FOTOC quantities are averaged identically). The regular and chaotic dynamics for the initial state $|\Psi _0^{\mathrm{c}}\rangle$ are clearly delineated: $\overline {S_2(\hat \rho _{{\mathrm{ph}}})} \approx 0$ for *B* > *B*_c_ and $\overline {S_2(\hat \rho _{{\mathrm{ph}}})} \ > \ 0$ for *B* < *B*_c_ respectively. Error bars indicate standard deviation of temporal fluctuations. In the inset we plot the same FOTOC quantities but including decoherence due to single-particle dephasing at the rate Γ = 60 s^−1^. The coherent parameters *g*, *B* and *δ* are enhanced by a factor of 16 compared to the main panel, as per ref. ^60^. **c** Time-averaged distribution functions (markers) for spin-projection *P*(*M*_z_) and phonon occupation *P*(*n*) (6 ms ≤ *t* ≤ 12 ms). We compare to the distribution of the diagonal ensemble (purple bars, see Methods). **d** Bipartite RE $S_2(\hat \rho _{L_A})$ (black markers) as a function of partition size *L*_*A*_ of the spins, averaged over same time window as (**c**). For comparison, we plot the RE of a thermal canonical ensemble with corresponding temperature *T* fixed by the energy of the initial state $|\Psi _0^{\mathrm{c}}\rangle$, $S_2^{{\mathrm{therm}}}$ and the RE of the diagonal ensemble (see Methods). Volume-law behavior of the RE is replicated by the FOTOC quantity (blue markers). Note that the dimension of the spin Hilbert space scales linearly with *L*_*A*_. Shaded regions indicate standard deviation of temporal fluctuations. Data for (**a**)–(**d**) is obtained for *N* = 40, with *g* and *δ* identical to calculations of Fig. 2. For (**c**) and (**d**) we choose *B*/(2*π*) = 0.7 kHz (*B*/*B*_c_ = 0.2). Source data are provided as a Source Data file

In Fig. 4a we show the typical behavior of the RE, $S_2(\hat \rho _{{\mathrm{ph}}})$, in the two different phases for |Ψ_0_〉. First, in the normal phase (panel (i)), *B* ≪ *B*_c_, the dynamics is dominated by precession about the transverse field and the entanglement entropy exhibits small amplitude oscillations^53^. Conversely, in the superradiant phase (panel (ii)) *B* ≪ *B*_c_ we observe a rapid growth of entanglement and saturation past the transient regime. We summarize our results in Fig. 4b where we plot the time-averaged value of $S_2\left( {\hat \rho _{{\mathrm{ph}}}} \right)$ vs. $\sqrt {B_{\mathrm{c}}/B}$. We associate the fast growth of $S_2\left( {\hat \rho _{{\mathrm{ph}}}} \right)$ at $B/B_{\mathrm{c}}\sim 1$ with a crossover from the integrable to the chaotic regime. To further illustrate this connection, we compare the approximate RE obtained via $S_{\mathrm{F}}^{\hat S_{\mathbf{r}},\hat n} \equiv - {\mathrm{log}}[I_0^{\hat S_{\mathbf{r}}}(t) + I_0^{\hat n}(t)]$ and $S_{\mathrm{F}}^{\hat S_{\mathbf{r}}} \equiv - {\mathrm{log}}[I_0^{\hat S_{\mathbf{r}}}]$ with the exact RE in Fig. 4b. It is observed that in all parameter regimes one can make a quantitative link between the RE and FOTOCs, especially under proper optimization of the rotation axis $\hat S_{\mathbf{r}}$ at each time to minimize the coherence and diagonal terms in Eq. (4) (see Methods and Supplementary Methods).

The saturation of $S_2(\hat \rho _{{\mathrm{ph}}})$ for *B* < *B*_c_ is a signature of thermalization. One can test how “thermalized” the quantum system is by comparing the behavior of the spin and phonon distributions in the long time limit with those of the corresponding diagonal ensemble, characterized by a mixed density matrix $\hat \rho _{\mathrm{D}}$ with purely diagonal elements (see Methods)^1–3^. These comparisons are shown in Fig. 4c, where the time evolved distributions and the ones drawn from the diagonal ensemble are almost indistinguishable.

We can also investigate the growth of entanglement on different size bipartitions for *B* < *B*_c_. For that we split the spin system into a subsystem of size *L*_*A*_ ≤ *N* and evaluate $S_2(\hat \rho _{L_A})$ by computing the reduced density matrix $\hat \rho _{L_A}$ by tracing over the bosonic degree of freedom and the remaining *N* − *L*_*A*_ spins. To demonstrate the entanglement grows with system size in a manner consistent with an equivalent thermal state we plot the predictions of a canonical ensemble (see Methods). We observe volume-law entanglement growth for *L*_*A*_ ≪ *N* (see Fig. 4d). However, for *L*_*A*_ *~* *N* the entanglement growth deviates from this simple prediction. These deviations occur as the full state of the system is pure, and thus eventually one needs to recover $S_2(\hat \rho ) = 0$, requiring a negative curvature. To demonstrate the intertwined nature of thermalization and the buildup of entanglement we plot the predictions of a canonical ensemble indicated by the dotted purple line (see Methods). We note that FOTOCs can also be used to probe this scaling of the RE with subsystem size. To this end, both $\hat V$ and $\hat W$ should be restricted to a partition of size *L*_*A*_ of the system, but otherwise the corresponding multiple quantum intensities are computed as discussed above (see also Methods). Figure 4d shows the excellent agreement between the partial system FOTOCs (blue squares) and RE (black diamonds), comparisons that illustrate the utility of FOTOCs to characterize complex many-body entanglement.

### Experimental implementation in trapped ion simulators

Trapped ions present a promising experimental platform for the investigation of the physics discussed here^27,54,55^. Here we focus on two-dimensional arrays in a Penning trap where a tunable coupling between the ion’s spin, encoded in two hyperfine states, and the phononic center-of-mass (COM) mode of the crystal can be implemented by a pair of lasers with a beatnote frequency detuned by *δ* from the COM mode and far from resonance to all other modes, which remain unexcited (Fig. 1b). In the presence of microwaves (which generate the transverse field) resonant with the spin level splitting, the effective Hamiltonian is of the form of Eq. 2 as benchmarked in refs. ^27,56^. The dynamical control of the transverse field and sign of the detuning from the COM mode enables straightforward implementation of a time-reversal protocol to measure FOTOCs^19^ (see Fig. 1). Additionally, the many-body echo requires the application of a spin echo *π* pulse along $e_r = \hat y$ which reverses the signs of $\hat S_x$ and $\hat S_z$ simultaneously.

Our proposal requires the ability for measuring the fidelity of the full spin−phonon state, which we have not yet demonstrated experimentally. However, this will be possible through a generalization of the protocol discussed in ref. ^57^ (see Methods). Additionally, our proposal can be adversely affected by decoherence present in the experiment. However, the impact of decoherence will be minimized in future experiments by increasing the magnitude of relevant couplings of the DM via parametric amplification of the ions’ motion^27,58^, thus reducing the ratio of dissipative to coherent evolution. We illustrate the predicted effect of decoherence, which is dominated by single-particle dephasing due to light scattering from the lasers, in the inset of Fig. 4b. We include the enhancement of the coherent parameters via the protocol described in ref. ^58^ while using the typical experimental decoherence rate of Γ = 60 s^−1^. The single-particle decoherence is modeled by an exponential decay of the FOTOC components $I_0^{\hat G} \to I_0^{\hat G}e^{ - \Gamma Nt}$ (see Methods). The numerical calculation indicates that even with decoherence the crossover between the two regimes at *B* ~ *B*_c_ is still well captured. Due to numerical complexity of solving a master equation we restrict our simulations to *N* = 40 ions.

## Discussion

We have demonstrated that FOTOCs connect the fundamental concepts of scrambling, chaos, quantum thermalization, and multipartite entanglement in the DM. While the concepts presented here have been limited to collective Hamiltonians, we believe they can be generalized to more complex many-body models (Supplementary Note 2). For example, FOTOCs could provide an alternative approach for performing efficient measurements of RE in a way comparable to other state-of-the-art methods which have been used to probe entanglement in systems with up to 20 ions^7^. Generically, FOTOCs could serve as an experimental tool capable of uncovering bounds on information transport and computational complexity, and shed light on how classical behaviors in macroscopic systems emerge from purely microscopic quantum effects.

*Note added***:** Upon completion of this manuscript we became aware of the recent preprints^59,60^, which present the numerical and analytic investigation of OTOCs in the Dicke model.

## Methods

### Classical dynamics and equations of motion

The results presented for the classical model in Fig. 3 are obtained from the Heisenberg equations of motion for the operators via a mean-field ansatz, wherein the operators are replaced by the c-number expectation values, i.e., $\hat S_j \to \langle \hat S_j\rangle$ for $j = x,y,z$ and $\hat a \to \langle \hat a\rangle$ (where we adopt $\alpha _{{\mathrm{R(I)}}}$ as the real (imaginary) component of $\langle \hat a\rangle$). We thus obtain an equation of motion for ${\tilde{\mathbf{x}}} = (\langle \hat S_x\rangle ,\langle \hat S_y\rangle ,\langle \hat S_z\rangle ,\alpha _{\mathrm{R}},\alpha _{\mathrm{I}})$,

5 $$\frac{{{\mathrm{d}}{\tilde{\mathbf{x}}}}}{{{\mathrm{d}}t}} = F({\tilde{\mathbf{x}}}),$$

where

6 $$F({\tilde{\mathbf{x}}}) = \left( {\begin{array}{*{20}{c}} { - \delta \alpha _{\mathrm{I}}} \\ {\delta \alpha _{\mathrm{R}} - \frac{{2g}}{{\sqrt N }}\langle \hat S_z\rangle } \\ { - \frac{{4g}}{{\sqrt N }}\alpha _{\mathrm{R}}\langle \hat S_y\rangle } \\ { - B\langle \hat S_z \rangle+ \frac{{4g}}{{\sqrt N }}\alpha _{\mathrm{R}}\langle \hat S_x\rangle } \\ {B\langle \hat S_y\rangle } \end{array}} \right).$$

### Lyapunov exponent

The existence of classical chaos can be characterized by the Lyapunov exponent *λ*_L_. By definition, classical chaos implies that two initially close trajectories separated by a distance in phase-space $\Delta {\tilde{\mathbf{x}}}(0) = |{\tilde{\mathbf{x}}}_1(0) - {\tilde{\mathbf{x}}}_2(0)|$ diverge exponentially, $|\Delta {\tilde{\mathbf{x}}}(t)| \approx |\Delta {\tilde{\mathbf{x}}}(0)|e^{\lambda _{\mathrm{L}}t}$, and thus *λ*_L_ > 0 is a signature of chaotic dynamics.

Formally, the Lyapunov exponent is then defined by taking the limit^36^

7 $$\lambda _{\mathrm{L}} \equiv \mathop {{\lim }}\limits_{t \to \infty } \mathop {{\lim }}\limits_{|\Delta {\tilde{\mathbf{x}}}(0)| \to 0} \frac{1}{t}{\mathrm{log}}\frac{{|\Delta {\tilde{\mathbf{x}}}(t)|}}{{|\Delta {\tilde{\mathbf{x}}}(0)|}}.$$

As the phase-space of our co-ordinate system is bounded, we evaluate Eq. (7) using the tangent-space method^35,61^. Essentially, rather than monitoring the physical separation $|\Delta {\tilde{\mathbf{x}}}(t)|$ of a pair of initially nearby trajectories, one can instead solve for the separation in tangent space, denoted by $\delta {\tilde{\mathbf{x}}}(t)$, and substitute this distance into Eq. (7). The tangent-space separation $\delta {\tilde{\mathbf{x}}}(t)$ can be dynamically computed by assuming an infinitesimal initial perturbation to a reference trajectory starting at ${\tilde{\mathbf{x}}}(0) = {\tilde{\mathbf{x}}}_0$, leading to the system of equations

8 $$\frac{{{\mathrm{d}}{\tilde{\mathbf{x}}}}}{{{\mathrm{d}}t}} = F({\tilde{\mathbf{x}}}),$$

9 $$\frac{{{\mathrm{d}}{\mathbf{\Phi}}}}{{{\mathrm{d}}t}} = {\mathbf{M}}{{\Phi}}.$$

Here, **Φ** is the fundamental matrix and $M_{ij} \equiv {\mathrm{d}}F_i/{\mathrm{d}}x_j$. The tangent-space separation with respect to the initial point in phase-space ${\tilde{\mathbf{x}}}(0) = {\tilde{\mathbf{x}}}_0$ is extracted by computing $\delta {\tilde{\mathbf{x}}}(t) \equiv {\mathbf{\Phi}}\delta {\tilde{\mathbf{x}}}(0)$ with ${\mathbf{\Phi}}(0) = {\mathbb{I}}$.

As we are only interested in the maximum Lyapunov exponent, it suffices to choose the initial separation $\delta {\tilde{\mathbf{x}}}(0)$ along a random direction in phase-space, and we propagate Eqs. (8) and (9) for each initial condition ${\tilde{\mathbf{x}}}_0$ for sufficiently large *t* that our estimate of *λ*_L_ from Eq. (7) converges.

### Connection between classical and quantum Lyapunov exponents

In our discussion of the exponential growth of FOTOCs, we have argued that *λ*_Q_ is intimately related to the classical Lyapunov exponent *λ*_L_. Specifically, we have that $\lambda _{\mathrm{Q}} \simeq 2\lambda _{\mathrm{L}}$. Here, we further articulate this connection using a semi-classical description of the quantum dynamics, specifically by considering the evolution in the truncated Wigner approximation (TWA)^39^.

First, we remind the reader that for a small perturbation *δϕ*, a FOTOC ${\cal{F}}_G(t,\delta \phi )$ can be expanded to ${\cal{O}}(\delta \phi ^2)$ as ${\cal{F}}_G(t,\delta \phi ) \approx 1 - \delta \phi ^2{\mathrm{var}}(\hat G)$. A simple conclusion from this expansion is that if ${\cal{F}}_G(t,\delta \phi )$ grows exponentially we can attribute this behavior to the variance, i.e. it must be true that ${\mathrm{var}}(\hat G)\rangle \sim e^{\lambda _{\mathrm{Q}}t}$.

A semi-classical explanation of this exponential growth is simplified by assuming that $\hat G$ is an operator which is linear in the classical phase-space variables ${\tilde{\mathbf{x}}}$. For concreteness, let us consider $\hat G = \hat X = \frac{1}{2}(\hat a + \hat a^\dagger )$ as in Fig. 3 of the main text, which corresponds to *α*_R_ in the classical phase-space.

Next, we consider a description of the quantum dynamics within the framework of the TWA. Here, the dynamics is computed by solving the classical equations of motion (Eq. (5)) with random initial conditions sampled from the corresponding Wigner phase-space distribution of the initial state^39^. Quantum expectation values are then obtained by appropriate averaging over an ensemble of trajectories, e.g., $\langle \hat X\rangle \equiv \overline {\alpha _{\mathrm{R}}}$ where the overline denotes a stochastic average. The random sampling of initial conditions serves to model the quantum fluctuations of the initial state.

For a classically meaningful initial state (i.e. a product of coherent states for the phonon and spin degrees of freedom), the fluctuations in each of the phase-space variables are typically Gaussian and centered around the expectation values of the initial state. A concrete example to illustrate this is the state $|\Psi _0^{\mathrm{c}}\rangle = |( - N/2)_x\rangle \otimes |0\rangle$ considered in the main text. For each trajectory, the variable (*α*_R_)_*j*_ (*j* denoting the trajectory), for example, is sampled from a Gaussian distribution with mean zero and variance 1/4. The connection between the quantum dynamics and classical chaos is made by instead considering sampling only the fluctuations *δα*_R_ about a central classical trajectory, i.e. $(\alpha _{\mathrm{R}})_j \to \alpha _{\mathrm{R}}^{cl} + (\delta \alpha _{\mathrm{R}})_j$.

Solving the dynamics of the central classical trajectory and the ensemble of fluctuations is then identical to the calculation of Eqs. (8) and (9), from which the Lyapunov exponent is calculated. In particular, the connection between quantum and classical exponents is finally made clear by evaluating the quantum variance,

10 $${\mathrm{var}}(\hat X) = \left( {\overline {\alpha _{\mathrm{R}}^2} - \overline {\alpha _{\mathrm{R}}} ^2} \right),$$

11 $$\equiv \left( {\overline {\delta \alpha _{\mathrm{R}}^2} - \overline {\delta \alpha _{\mathrm{R}}} ^2} \right).$$

As *δα*_R_ is evaluated directly from Eq. (9), then we expect from our previous calculations that $|\delta \alpha _{\mathrm{R}}|\sim e^{\lambda _{\mathrm{L}}t}$ for a generic random perturbation, sampled according to the TWA prescription, in parameter regimes where there is classical chaos. Thus, we extrapolate that the quantum variance will grow like $\overline {\delta \alpha _{\mathrm{R}}^2} - \overline {\delta \alpha _{\mathrm{R}}} ^2\sim e^{2\lambda _{\mathrm{L}}t}$. Inspection of this final result shows that we should expect $\lambda _{\mathrm{Q}} \simeq 2\lambda _{\mathrm{L}}$.

### Connection between FOTOCs and RE

The connection between the FOTOCs and entanglement entropy is best established by first considering the case of the spin−phonon RE $S_2(\hat \rho _{{\mathrm{ph}}})$. We begin by writing the purity of the reduced density matrix explicitly in terms of the elements of the density matrix,

12 $${\mathrm{Tr}}\left[ {\hat \rho _{{\mathrm{ph}}}^2(t)} \right] = \mathop {\sum}\limits_{\scriptstyle n,n^\prime \atop\\ \scriptstyle m_{\mathbf{r}},m_{\mathbf{r}}^\prime } {\varrho _{m_{\mathbf{r}},m_{\mathbf{r}}}^{n,n^\prime }} (t)\varrho _{m_{\mathbf{r}}^\prime ,m_{\mathbf{r}}^\prime }^{n^\prime ,n}(t).$$

Our insight is that, in the case of a pure global state, the summation in Eq. (12) for the purity of the reduced density matrix can be manipulated and re-expressed as

13 $${\mathrm{Tr}}\left[ {\hat \rho _{{\mathrm{ph}}}^2(t)} \right] \equiv I_0^{\hat S_{\mathbf{r}}}(t) + I_0^{\hat n}(t) - D_{{\mathrm{diag}}}^{\hat S_{\mathbf{r}},\hat n}(t) + C_{{\mathrm{off}}}^{\hat S_{\mathbf{r}},\hat n}(t),$$

where

14 $$D_{{\mathrm{diag}}}^{\hat S_{\mathbf{r}},\hat n}(t) = \mathop {\sum}\limits_{\scriptstyle n,\atop\\ \scriptstyle m_{\mathbf{r}}} {\left[ {\rho _{m_{\mathbf{r}},m_{\mathbf{r}}}^{n,n}(t)} \right]^2} ,$$

15 $$C_{{\mathrm{off}}}^{\hat S_{\mathbf{r}},\hat n}(t) = \mathop {\sum}\limits_{\scriptstyle n \ne n\prime \atop\\ \scriptstyle m_{\mathbf{r}} \ne m_{\mathbf{r}}^\prime } {\rho _{m_{\mathbf{r}},m_{\mathbf{r}}}^{n,n\prime }} (t)\rho _{m_{\mathbf{r}}^\prime ,m_{\mathbf{r}}^\prime }^{n\prime ,n}(t),$$

are the sum of the squared diagonal elements of the density matrix and the sum over the off-diagonal coherences, respectively. Thus, we seek to understand when these latter terms can be neglected and thus the purity (and associated entropy) is expressible in terms of only the $I_0^{\hat G}$.

Firstly, there is the case of a large transverse field, $B \gg B_c$ and an initial state which is polarized along the direction of the transverse field with vacuum occupation, i.e., $|\Psi _0\rangle = |( \pm N/2)_x\rangle \otimes |0\rangle$. In this case, we expect the collective spin to remain strongly polarized along the field direction. If we choose the FOTOC spin rotation axis to be along that of the initial state and transverse field, $\hat S_{\mathbf{r}} = \hat S_x$, then we have that $C_{{\mathrm{off}}}^{\hat S_x,\hat n}(t) \approx 0$ due to the absence of initial coherences between the spin sectors in this basis, and by similar reasoning $I_0^{\hat n}(t) \approx D_{{\mathrm{diag}}}^{\hat S_x,\hat n}(t)$. Hence, we expect Eq. (13) to simplify so that ${\mathrm{Tr}}[\hat \rho _{{\mathrm{ph}}}^2(t)] \approx I_0^{\hat S_x}(t)$. Identical reasoning can be applied in the normal phase (*B* > *B*_c_) when the phonon detuning is the largest energy scale, such that ${\mathrm{Tr}}[\hat \rho _{{\mathrm{ph}}}^2(t)] \approx I_0^{\hat n}(t)$.

The second scenario is closely related to the first. Consider an initial coherent spin state polarized along an arbitrary spin direction and vacuum phonon occupation. For arbitrary transverse field strength and on sufficiently short time-scales $t \lesssim \lambda _{\mathrm{Q}}^{ - 1}$, then the spin component of the evolved state remains largely polarized along a particular axis dictated by the initial state. Similar to the first scenario, by choosing the spin rotation of the FOTOC, $\hat S_{\mathbf{r}}$, to match the polarization of the initial state, then we will have ${\mathrm{Tr}}[\hat \rho _{{\mathrm{ph}}}^2(t)] \approx I_0^{\hat S_{\mathbf{r}}^2}(t)$. This is justified as $C_{{\mathrm{off}}}^{\hat S_{\mathbf{r}},\hat n}(t)$ and $I_0^{\hat n}(t) + D_{{\mathrm{diag}}}^{\hat S_{\mathbf{r}},\hat n}(t)$ vanish, as again the state at short times will not have appreciable coherences between different spin sectors in this basis.

Lastly, for a small transverse field, $B \ll B_c$, and beyond short times $t \ \gtrsim \ \lambda _{\mathrm{Q}}^{ - 1}$ (i.e., beyond the time-scale when the spin state is still strongly polarized and the second scenario is still valid), we expect Eq. (13) to be well approximated by ${\mathrm{Tr}}[\hat \rho _{{\mathrm{ph}}}^2(t)] \approx I_0^{\hat S_{\mathbf{r}}}(t) + I_0^{\hat n}(t)$ for any spin rotation axis $\hat S_{\mathbf{r}}$. This is because initially pure states which are sufficiently scrambled after a quench of the system parameters closely resemble so-called canonical pure thermal quantum (cTPQ) states^62^ in a generic basis. For cTPQ states, the summation over off-diagonal coherences $C_{{\mathrm{off}}}^{\hat S_{\mathbf{r}},\hat n}$ vanishes exactly for a sufficiently large system as the coherences can be considered as random variables^62^. Moreover, for a typical spin rotation axis $\hat S_{\mathbf{r}}$, the cTPQ state will have a spin distribution $P(M_{\hat S_{\mathbf{r}}})$ which is largely delocalized implying that $D_{{\mathrm{diag}}}^{\hat S_{\mathbf{r}},\hat n}\sim 1/(Nn_{{\mathrm{ph}}})$ where *n*_ph_ is some constant which characterizes the spread of the boson number distribution. The term $D_{{\mathrm{diag}}}^{\hat S_{\mathbf{r}},\hat n}$ is then typically much smaller in magnitude when compared to the remaining terms $I_0^{\hat S_{\mathbf{r}}}$ and $I_0^{\hat n}$. This reasoning leads to ${\mathrm{Tr}}[\hat \rho _{{\mathrm{ph}}}^2(t)] \approx I_0^{\hat S_{\mathbf{r}}}(t) + I_0^{\hat n}(t)$. Discussion of the sensitivity of these arguments to the rotation direction can be found in Supplementary Methods.

More generally, we can extend these arguments to extract a correspondence with the Renyi entropy of a generic bipartition of the spin−phonon system. Specifically, splitting the system ${\cal{S}}$ into a subsystem *A*: *L* spin-1/2s, and its complement *A*_*c*_: *N* − *L* spin-1/2s and the bosonic mode. In the weak-field regime $B \ll B_c$, ${\mathrm{Tr}}[\hat \rho _A^2] \approx I_0^A + I_0^{A_c}$. Here, the terms $I_0^A$ and $I_0^{A_c}$ are obtained as the Fourier amplitudes of fidelity OTOCs for generalized rotations within each subsystem. Specifically, a local rotation $e^{i\phi \hat S_{{\mathbf{r}},A}}$ taken to act on the spin-12s in the *A* subsystem, and a joint (but uncorrelated) rotation $e^{i\phi \hat S_{{\mathbf{r}},A_c}}e^{i\theta \hat a^\dagger \hat a}$ of the spins and bosons in the complement *A*_*c*_.

### Experimental implementation

By preparing an initial spin polarized state, recent experiments^18^ demonstrated it was possible to measure the many-body overlap of the final state with the initial configuration by fluorescence detection. The Dicke model, however, includes spin and phonon degrees of freedom.

While the full spin–phonon fidelity measurement has not yet been demonstrated experimentally, such measurement is possible by extending the method in^57^ to a multi-qubit system. In particular, we note that this proposal is comprised of a two-step measurement, where we first measure the spin degree of freedom. The probability of all ions being in the dark state (i.e. all in the state |↓〉_*z*_) can be measured with excellent fidelity and has been previously demonstrated^18^. The dark state does not scatter photons, and as such, this measurement will not change the state of the phonons. Next one can proceed to measure the phonon occupation via the protocol described in ref. ^57^.

Finally, as noted in the main text, we have taken into account the single-particle decoherence present in the experiment. The results presented in the main text accounted for this by approximating the effects of decoherence by an exponential decay, $\bar I_0^{\hat G} \to \bar I_0^{\hat G}e^{ - \Gamma Nt}$. We have justified this approximation by comparing to an efficient numerical solution of the full Lindblad master equation^19,63,64^ for smaller system sizes (*N* = 10). We find that the decoherence is well-captured by the approximate model for all transverse field strengths *B* considered.

### Thermal and diagonal ensembles

The canonical thermal ensemble, used in Fig. 4, is defined by the density matrix $\hat \rho _{{\mathrm{therm}}} = e^{ - \beta \hat {\mathrm{H}}_{\mathrm{D}}}/{\mathrm{Tr}}[e^{ - \beta \hat {\mathrm{H}}_{\mathrm{D}}}]$, which is characterized by the inverse temperature *β* = 1/(*k*_B_*T*). This inverse temperature is chosen such that energy of the ensemble is matched to that of the initial state of the dynamics, $\langle E\rangle _{{\mathrm{therm}}} \equiv {\mathrm{Tr}}[\hat {{\mathrm{H}}}_{\mathrm{D}}\hat \rho _{{\mathrm{therm}}}] = \langle \Psi _0|\hat {\mathrm{H}}_{\mathrm{D}}|\Psi _0\rangle$. The RE for bipartitions of the thermal ensemble is then obtained via the definition $S_2^{{\mathrm{therm}}} \equiv - {\mathrm{log}}\left( {{\mathrm{Tr}}[(\hat \rho _{L_A}^{{\mathrm{therm}}})^2]} \right)$ where $\hat \rho _{L_A}^{{\mathrm{therm}}} = {\mathrm{Tr}}_{{\mathrm{ph}},N - L_A}(\hat \rho _{{\mathrm{therm}}})$ is the reduced density matrix obtained after tracing out the phonon degree of freedom and the remaining *N* − *L*_*A*_ spins.

A related concept is the diagonal ensemble $\hat \rho _{\mathrm{D}}$^1,65^, which generically describes the (time-averaged) observables of a quantum system which has relaxed at long times. The ensemble is defined as the mixed state $\hat \rho _{\mathrm{D}} \equiv \mathop {\sum}\nolimits_{E_n} |c_{E_n}|^2|E_n\rangle \langle E_n|,$ where $c_{E_n} \equiv \langle \Psi _0|E_n\rangle$ and |*E*_*n*_〉 are the eigenstates of the Hamiltonian $\hat {\mathrm{H}}_{\mathrm{D}}$ with associated eigenvalue *E*_*n*_. We use this diagonal ensemble as a comparison to the time-averaged distribution functions *P*(*M*_z_) and *P*(*n*) in Fig. 4.

## Supplementary information

Supplementary Information

## Data Availability

The source data underlying Figs. 2–4 of the main text are provided as a source data file. Additional numerical data and computer codes used in this study are available from the corresponding author upon request.
