# Author Correction: Unifying scrambling, thermalization and entanglement through measurement of fidelity out-of-time-order correlators in the Dicke model

**DOI:** 10.1038/s41467-019-13016-5

**Published:** 2019-10-29

**Authors:** R. J. Lewis-Swan, A. Safavi-Naini, J. J. Bollinger, A. M. Rey

**Affiliations:** 1 JILA, NIST and Department of Physics, University of Colorado, Boulder, CO 80309 USA; 2 Center for Theory of Quantum Matter, University of Colorado, Boulder, CO 80309 USA; 3 NIST, Boulder, CO 80305 USA

**Keywords:** Quantum information, Quantum simulation, Statistical physics, thermodynamics and nonlinear dynamics

Correction to: *Nature Communications* 10.1038/s41467-019-09436-y, published online 5 April 2019.

The original version of this Article omitted references to previous work in ‘Fine, B. V., Elsayed, T. A., Kropf, C. M. & de Wijn, A. S. Absence of exponential sensitivity to small perturbations in nonintegrable systems of spins 1/2. *Phys. Rev. E* **89**, 012923 (2014)’ and ‘Elsayed, T. A. & Fine, B. V. Sensitivity to small perturbations in systems of large quantum spins. *Phys. Scr*. **2015**, 014011 (2015)’. These have been added as references 64 and 65 at the end of the penultimate sentence of the last paragraph of the ‘Connections between scrambling dynamics and chaos’ section of the Results: ‘A similar factor of two relating the classical and quantum exponents has previously been observed in refs. ^37,47,64,65^.’ This has been corrected in the PDF and HTML versions of the Article.

