# Supplementary material for: Unifying scrambling, thermalization and entanglement through measurement of fidelity out-of-time-order correlators in the Dicke model
Source: Nat Commun. 2019 Apr 5;10:1581. doi: 10.1038/s41467-019-09436-y (PMC6450886; doi:10.1038/s41467-019-09436-y)
Supplement: Supplementary file 1 — Supplementary Information [file 41467_2019_9436_MOESM1_ESM.pdf]

Supplemental Information: Unifying scrambling, thermalization and entanglement through the measurement of fidelity out-of-time-order correlators in the Dicke model

R. J. Lewis-Swan,[1,2] A. Safavi-Naini,[1,2] J. J. Bollinger,[3] and A. M. Rey[1,2]

[1]*JILA, NIST and Department of Physics, University of Colorado, Boulder, USA*
[2]*Center for Theory of Quantum Matter, University of Colorado, Boulder, CO 80309, USA*
[3]*NIST, Boulder, CO 80305, USA*

(Dated: February 28, 2019)

Supplementary Methods

In the main text we leave unspecified the exact form of the time-dependent spin-rotation $\hat{S}_{\mathbf{r}}$ from which the MQC $I_0^{\hat{S}_{\mathbf{r}}}(t)$ is obtained in, e.g. Fig. 4 of the main text, although we have argued that any generic rotation should yield a good approximation to the Renyi entropy. However, for the still relatively small systems we consider it is clear that there will exist an 'optimal' rotation choice for which our arguments will give the best quantitative correspondence, i.e. a basis in which the state appears the 'most scrambled'. By this we mean that when the density matrix is written in the basis of the optimal rotation $\hat{S}_{\mathbf{r}}$, the state has a broad spin probability distribution $P(M_{\mathbf{r}})$ and there are a large number of essentially random off-diagonal coherences which respectively lead to a suppression of $D_{\mathrm{diag}}^{\hat{S}_{\mathbf{r}},\hat{n}}$ and $C_{\mathrm{off}}^{\hat{S}_{\mathbf{r}},\hat{n}}$.

The (time-dependent) choice of $\hat{S}_{\mathbf{r}}$ which guarantees this is not neccesarily clear *a priori*. However, in Supplementary Fig. 1 we demonstrate that: i) For sufficiently scrambled states of the Dicke model, any choice of $\hat{S}_{\mathbf{r}}$ gives a qualitatively robust correspondence to the entanglement entropy, and ii) an educated guess for $\hat{S}_{\mathbf{r}}$ can be made without rigourously optimising the FOTOCs over all rotation axes, but rather by searching for the maximum variance $\mathrm{var}(\hat{S}_{\mathbf{r}})$ after time t (i.e., the first half of the many-body echo sequence). The latter can be understood to be a crude measure of how delocalized the distribution $P(M_{\mathbf{r}})$ is. We do reiterate, however, that the results plotted in Supplementary Fig. 1 clearly show that any rotation axis does always track the qualitative and indeed quantitative structure of the full Renyi entropy, and the optimisation is thus only fine-tuning.

Supplementary Note 1

In the main text we present data for λ_{Q} and λ_{L} in Supplementary Fig. 3 obtained from sample initial states $|\Psi_0^c\rangle = |-N/2\rangle_x \otimes |0\rangle$ and $|\Psi_0^y\rangle = |-N/2\rangle_y \otimes |0\rangle$ as a function of B/B_c and the FOTOC $\mathcal{F}_X(t)$. The results plotted in Supplementary Fig. 3 validate our predicted relation $\lambda_{\mathrm{Q}} \simeq 2\lambda_{\mathrm{L}}$.

Here, we elaborate on this data in two ways. First, we show the generic growth of the FOTOC for not only the exemplary state $|\Psi_0^c\rangle$ (also shown in Fig. 3 of the main text) but also $|\Psi_0^y\rangle$. These results are plotted in Supplementary Fig. 2 and demonstrate that generically, we do not observe perfect exponential growth, as for the state $|\Psi_0^c\rangle$, but rather an oscillatory function which grows with an exponential trend. We plot against $\sim e^{2\lambda_{\mathrm{L}} t}$ for comparison. The exemplary nature of the exponential growth for $|\Psi_0^c\rangle$ is attributable to it being an unstable fixed point of the classical phase-space with $\langle \hat{X} \rangle = \overline{\alpha_R} = 0$.

Second, we also demonstrate the broad validity of our relation between the quantum and classical exponents for other FOTOCs/operators. Specifically, we plot the growth of $\mathcal{F}_{S_y}(t)$ and $\mathcal{F}_n(t)$. For $\mathcal{F}_{S_y}(t)$, we observe exponential growth similar to the results of $\mathcal{F}_X(t)$ such that $\mathcal{F}_{S_y}(t) \sim e^{\lambda_{\mathrm{Q}} t}$ and $\lambda_Q \approx 2\lambda_{\mathrm{L}}$ as previous. However, the results for $\mathcal{F}_n(t)$ require some further explanation. Specifically, $\hat{n} = \hat{a}^\dagger \hat{a}$ is nonlinear in the classical variables, i.e. $\hat{n} \to n \equiv \alpha_R^2 + \alpha_I^2$. Whilst in the classical model the seperation of trajectories measured in terms of the nonlinear variable n will still grow exponentially, they do so with a

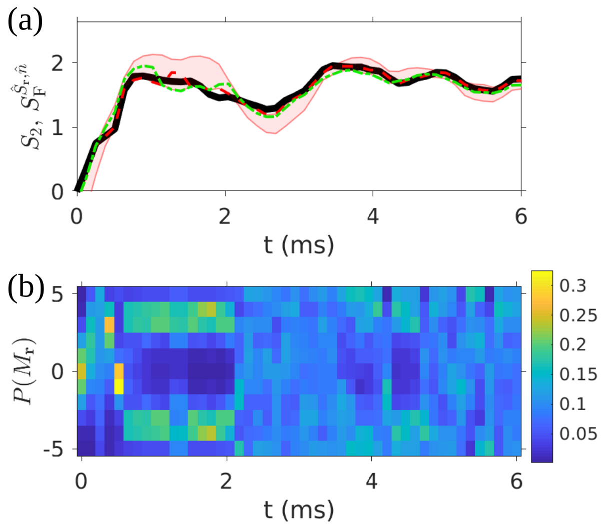

Supplementary Figure 1. Optimisation of FOTOC rotation axis. (a) Typical evolution of spin-phonon entanglement $\hat{S}_2(\hat{\rho}_{\mathrm{ph}})$ (black solid line) in the chaotic phase. We find excellent agreement with the FOTOC quantity $S_{\mathrm{F}}^{\hat{S}_{\mathbf{r}},\hat{n}}$ for both rigorous optimisation of $\hat{S}_{\mathbf{r}}$ (red dashed line), optimisation via maximisation of $\mathrm{var}(\hat{S}_{\mathbf{r}})$ (green dot-dashed line). Shaded red regions represent full range of possible $S_{\mathrm{F}}^{\hat{S}_{\mathbf{r}},\hat{n}}$ for any rotation axis. (b) Spin probability distribution $P(M_{\mathbf{r}})$ for optimal rotation $\hat{S}_{\mathbf{r}}$ [red dashed line in (a)]. For $t \gtrsim 2$ ms the distribution becomes delocalized, consistent with the arguments in text. Data for both panels is for $B/B_c \approx 0.1$, $N = 10$ and all other parameters as per Fig. 4 of the main text. Source data are provided as a Source Data file.

different classical exponent: $|n_1(t) - n_2(t)| \approx |n_1(0) - n_2(0)|e^{\lambda_c t}$. As n is not an invertible transformation of the co-ordinates α_R and α_I then this classical exponent λ_{c} is not neccesarily identical to the Lyapunov exponent λ_{L} determined from the distance in terms of the natural phase-space variables. Indeed, for n we typically observe $\lambda_{\mathrm{c}} \approx 2\lambda_{\mathrm{L}}$ (see Supplementary Fig. 3). The key consequence of this subtlety is that while we still observe exponential growth of the FOTOC $\mathcal{F}_n(t) \sim e^{\lambda_{\mathrm{Q}}' t}$, we have that $\lambda_{\mathrm{Q}}' \approx 2\lambda_{\mathrm{c}}$ where λ_{Q}' is thus not neccesarily identical to λ_Q obtained from $\mathcal{F}_X(t)$ (or other FOTOCs formed from linear combinations of the phase-space co-ordinates). Thus, a more general statement of our finding relating the quantum and Lyapunov exponents in the main text is: the quantum exponent of an exponentially growing FOTOC is approximately twice that of the appropriately defined classical exponent, $\lambda_{\mathrm{Q}} = 2\lambda_{\mathrm{c}}$. In the case where the FOTOC operator $\hat{G}$ corresponds to a linear combination of the phase-space co-ordinates this reduces to $\lambda_{\mathrm{Q}} = 2\lambda_{\mathrm{L}}$ as $\lambda_{\mathrm{c}} \equiv \lambda_{\mathrm{L}}$. This result is reflected in the results plotted in Supplementary Fig. 2.

Supplementary Note 2

Here we outline a generalization of FOTOCs and the related multiple quantum intensities for a generic system. In particular, we consider how FOTOCs might be implemented for spin models which are not collective. We highlight, however, that our analysis can be straightforwardly generalized to other systems, such as bosonic models involving many modes (which appear in, e.g., quantum gas microscope experiments). Our analysis is closely related to that presented in pages 7-8 of the manuscript and the methods.

Let us consider a generic system $\mathcal{S}$ of N spin-$1/2$s, which can be arbitrarily split into two subsystems $\mathcal{A}$ and $\mathcal{B}$ containing $N_{\mathcal{A}}$ and $N_{\mathcal{B}}$ spins respectively. The system evolves under an arbitrary (but non-collective) Hamiltonian $\hat{H}$. We can write generic pure states of the system in terms of the basis $\{|\vec{a}\rangle\}$ and $\{|\vec{b}\rangle\}$, which are defined to be the tensor product of single particle states such that $|\vec{a}\rangle \equiv \bigotimes_j |a_j^{\mathbf{r}_j}\rangle$ (and similarly for $|\vec{b}\rangle$) where $\hat{\sigma}_j^{\mathbf{r}_j}|a_j^{\mathbf{r}_j}\rangle \equiv \pm|a_j^{\mathbf{r}_j}\rangle$ are eigenstates of the spin-projection along an arbitrary direction $\mathbf{r}_j$ on the

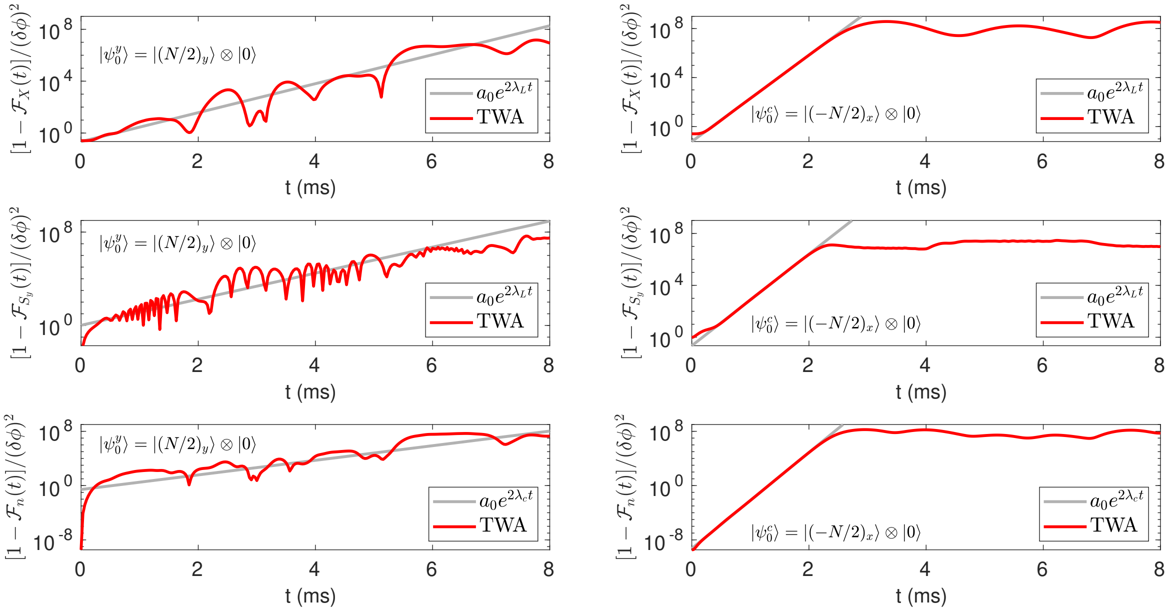

Supplementary Figure 2. Exponential growth of quantum variances $\mathrm{var}(\hat{X}) \approx [1-\mathcal{F}_X(t)]/(\delta\phi)^2$, $\mathrm{var}(\hat{S}_y) \approx [1-\mathcal{F}_{S_y}(t)]/(\delta\phi)^2$ and $\mathrm{var}(\hat{n}) \approx [1-\mathcal{F}_n(t)]/(\delta\phi)^2$ (red lines) for $N = 10^8$ from truncated Wigner calculations (assuming $\delta\phi \ll 1/N$). We give examples for $|\Psi_0^c\rangle = |(-N/2)_x\rangle \otimes |0\rangle$ (right) and $|\Psi_0^y\rangle = |(N/2)_y\rangle \otimes |0\rangle$ (left). Grey lines indicate a comparison to $a_0 e^{2\lambda_{L,c} t}$, with a_0 fitted to FOTOC data. Other parameters are same as calculations in Fig. 3a of the main text. Source data are provided as a Source Data file.

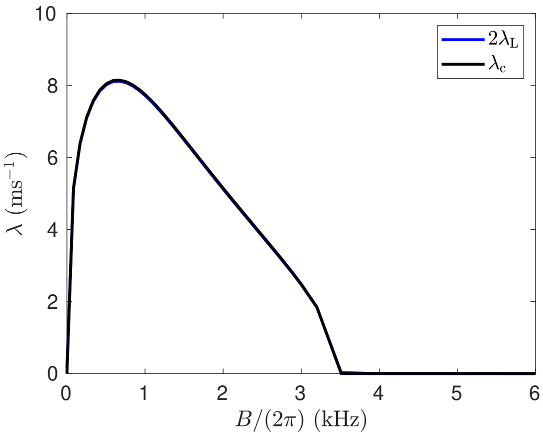

Supplementary Figure 3. Comparison of classical Lyapunov exponent λ_L and λ_c as a function of transverse field strength B/B_c. The latter is obtained by solution of the classical equations of motion (see Methods section of main text) and the definition $|n_1(t) - n_2(t)| \approx |n_1(0) - n_2(0)|e^{\lambda_c t}$ where the subscript labels two trajectories which are initially close in phase-space. The exponents are calculated for the initial condition $\tilde{\mathbf{x}} = (-N/2, 0, 0, 0, 0)$ corresponding to the quantum state $|\Psi_0^c\rangle$. Parameters are as per Figs. 2 and 3 of the main text. Source data are provided as a Source Data file.

spin-1/2 Bloch sphere for the jth qubit in $\mathcal{A}$. We emphasize that most generically the spin-projection is defined along an independent direction $\mathbf{r}_j$ for each of the qubits.

It is then straightforward to decompose the density matrix (similar to page 8 on the main text) in this

basis as:

$$\hat{\rho} = \sum_{\substack{\vec{a},\vec{a}' \\ \vec{b},\vec{b}'}} \varrho^{\vec{a},\vec{a}'}_{\vec{b},\vec{b}'} |\vec{a}\rangle\langle\vec{a}'| \otimes |\vec{b}\rangle\langle\vec{b}'|. \tag{1}$$

Similar to the collective model, we divide the density matrix into blocks of (single-particle) coherences $\hat{\rho} \equiv \sum_{\vec{M}} \hat{\rho}^{\{\hat{G}\}}_{\vec{M}}$ with respect to a set of single-particle operators $\{\hat{G}\}$. Specifically, we will focus on the case where $\{\hat{G}\} = \{\hat{\sigma}^{\mathbf{r}_j}_j | j \in \mathcal{A}\}$ or $\{\hat{G}\} = \{\hat{\sigma}^{\mathbf{r}_j}_j | j \in \mathcal{B}\}$ separately. For the former, we then define each block as

$$\hat{\rho}^{\{\hat{G}\}}_{\vec{M}} = \sum_{\substack{\vec{a}\in\mathcal{A} \\ \vec{b}\in\mathcal{B},\vec{b}'\in\mathcal{B}}} \varrho^{a_1^{\mathbf{r}_1}, a_2^{\mathbf{r}_2},\ldots,a_1^{\mathbf{r}_1}+M_1,a_2^{\mathbf{r}_2}+M_2,\ldots}_{b_1^{\mathbf{r}_1},b_2^{\mathbf{r}_2},\ldots,b_1^{\mathbf{r}_1},b_2^{\mathbf{r}_2},\ldots} \times \left[|a_1^{\mathbf{r}_1}\rangle\langle a_1^{\mathbf{r}_1} + M_1| \otimes |a_2^{\mathbf{r}_2}\rangle\langle a_2^{\mathbf{r}_2} + M_2| \otimes \ldots \otimes |b_1^{\mathbf{r}_1}\rangle\langle b_1^{\mathbf{r}_1}| \otimes |b_2^{\mathbf{r}_2}\rangle\langle b_2^{\mathbf{r}_2}| \otimes \ldots \right]. \tag{2}$$

Whilst this expression may look daunting, one can still define a set of generalized multiple quantum intensities $I^{\{\hat{G}\}}_{\vec{M}} \equiv \mathrm{Tr}\left[\hat{\rho}^{\{\hat{G}\}}_{\vec{M}} \hat{\rho}^{\{\hat{G}\}}_{-\vec{M}}\right]$, which are related to the generalized FOTOC $F_{\{\hat{G}\}}(t,\phi_1,\phi_2,\ldots) = \sum_{\vec{M}} I^{\{\hat{G}\}}_{\vec{M}} e^{-i\sum_j M_j\phi_j}$. This FOTOC is experimentally implemented by applying single-qubit rotations on the spins contained in $\mathcal{A}$ (alternately, $\mathcal{B}$) such that $\hat{W}_{\{\hat{G}\}} \equiv \bigotimes_{j\in\mathcal{A}} e^{i\phi_j\hat{\sigma}^{\mathbf{r}_j}_j}$. Here, the jth qubit is rotated about an independently chosen axis $\mathbf{r}_j$ by an angle ϕ_j.

Again, of most interest to us will be the 0-th multiple quantum intensity $I^{\{\hat{G}\}}_{\vec{0}} \equiv \mathrm{Tr}\,[(\,\hat{\rho}^{\{\hat{G}\}}_{\vec{0}}\,)^2]$ [i.e. $M_j = 0$ for all j in Eq. (2)]. This is accessed experimentally by performing a full set of OTOCs for a rangle of angles $\{\phi_j\}$, such that $I^{\{\hat{G}\}}_{\vec{0}} \propto \sum_{\{\phi_j\}} F_{\{\hat{G}\}}(t,\phi_1,\phi_2,\ldots)$. Similar to the main text, we can use this quantity to write the purity of the reduced density matrix in $\mathcal{A}$, $\hat{\rho}_{\mathcal{A}}$, as:

$$\mathrm{Tr}\left[\hat{\rho}^2_{\mathcal{A}}\right] \equiv I^{\{\hat{G}\}_{\mathcal{A}}}_{\vec{0}} + I^{\{\hat{G}\}_{\mathcal{B}}}_{\vec{0}} - D^{\{\hat{G}\}_{\mathcal{A}},\{\hat{G}\}_{\mathcal{B}}}_{\mathrm{diag}} + C^{\{\hat{G}\}_{\mathcal{A}},\{\hat{G}\}_{\mathcal{B}}}_{\mathrm{off}} \tag{3}$$

where $\{\hat{G}\}_{\mathcal{A}} \equiv \{\hat{\sigma}^{\mathbf{r}_j}_j | j \in \mathcal{A}\}$ and similar for $\{\hat{G}\}_{\mathcal{B}}$. The latter terms are given by

$$D^{\{\hat{G}\}_{\mathcal{A}},\{\hat{G}\}_{\mathcal{B}}}_{\mathrm{diag}} = \sum_{\vec{a},\vec{b}} \left[\varrho^{\vec{a},\vec{a}}_{\vec{b},\vec{b}}\right]^2, \tag{4}$$

and

$$C^{\{\hat{G}\}_{\mathcal{A}},\{\hat{G}\}_{\mathcal{B}}}_{\mathrm{off}} = \sum_{\substack{\vec{a}\neq\vec{a}' \\ \vec{b}\neq\vec{b}'}} \varrho^{\vec{a},\vec{a}'}_{\vec{b},\vec{b}}\, \varrho^{\vec{a}',\vec{a}}_{\vec{b}',\vec{b}'}. \tag{5}$$

Following the main text and methods, identical arguments can be made that the contributions from $D^{\{\hat{G}\}_{\mathcal{A}},\{\hat{G}\}_{\mathcal{B}}}_{\mathrm{diag}}$ and $C^{\{\hat{G}\}_{\mathcal{A}},\{\hat{G}\}_{\mathcal{B}}}_{\mathrm{off}}$ vanish in certain scenarios for a generic choice of operators $\{\hat{G}\}_{\mathcal{A},\mathcal{B}}$. In particular, the machinery of canonical pure thermal quantum (cTPQ) states can be used in the case of non-integrable systems to verify that $C^{\{\hat{G}\}_{\mathcal{A}},\{\hat{G}\}_{\mathcal{B}}}_{\mathrm{off}} \to 0$ for sufficiently large systems and $D^{\{\hat{G}\}_{\mathcal{A}},\{\hat{G}\}_{\mathcal{B}}}_{\mathrm{diag}} \ll I^{\{\hat{G}\}_{\mathcal{A}}}_{\vec{0}} + I^{\{\hat{G}\}_{\mathcal{B}}}_{\vec{0}}$ after short times.

The requirement of single-qubit rotations is similar in spirit to the protocol of random measurements proposed in Ref. [1], although the exact connection between the proposed schemes is an open question. We point out that alternatively one can also extract the required multiple quantum intensities by direct

measurement of joint probability distribution functions in the chosen bases:

$$I_{\vec{0}}^{\{\hat{G}\}_{\mathcal{A}}} \equiv \sum_{a\in\mathcal{A}} P(a_1^{\mathbf{r}_1}, a_2^{\mathbf{r}_2}, ...)^2, \tag{6}$$

$$\tag{7}$$

and similarly for $I_{\vec{0}}^{\{\hat{G}\}_{\mathcal{A}_c}}$. This form might be useful in, e.g., small chains of trapped ions and quantum gas microscope experiments (using a bosonic occupation basis), wherein one can measure such joint distributions relatively efficiently.

Lastly, we note that one can demonstrate that collective rotations of the spins in $\mathcal{A}$ and the associated $I_0^{\hat{S}_{\mathbf{r}}^{\mathcal{A}}}$ can be related to the equivalent $I_{\vec{0}}^{\{\hat{G}\}_{\mathcal{A}}}$ obtained via a uniform set of rotations with $\mathbf{r}_j = \mathbf{r}$. In particular, we have that the terms of $I_{\vec{0}}^{\{\hat{G}\}_{\mathcal{A}}}$ are contained within an appropriate expansion of $I_0^{\hat{S}_{\mathbf{r}}^{\mathcal{A}}}$ in the single-particle basis. This indicates that even for systems which span beyond the fully symmetric Dicke basis, collective rotations may allow us to gain some insight into the Renyi entropy. This is, however, an open question and currently under investigation.

1. Brydges, T. *et al.* Probing entanglement entropy via randomized measurements Preprint at https://arxiv.org/abs/1806.05747 (2018).
